# Silver Nanoparticles Biosynthesized Using *Achillea biebersteinii* Flower Extract: Apoptosis Induction in MCF-7 Cells via Caspase Activation and Regulation of Bax and Bcl-2 Gene Expression

**DOI:** 10.3390/molecules20022693

**Published:** 2015-02-05

**Authors:** Javad Baharara, Farideh Namvar, Tayebe Ramezani, Marzieh Mousavi, Rosfarizan Mohamad

**Affiliations:** 1 Research Center for Animal Development Applied Biology, Mashhad Branch, Islamic Azad University, Mashhad 917568, Iran; E-Mails: baharara@yahoo.com (J.B.); m_moosavi_k@yahoo.com (M.M.); 2 Department of Biology, Mashhad Branch, Islamic Azad University, Mashhad 917568, Iran; 3 Institute of Tropical Forestry and Forest Products (INTROP), Universiti Putra Malaysia, UPM Serdang, Selangor 43400, Malaysia; E-Mail: farizanmohd@gmail.com; 4 Faculty of Biological Sciences, Kharazmi University, Tehran 14911, Iran; E-Mail: tayeberamezani@gmail.com; 5 Department of Bioprocess Technology, Faculty of Biotechnology and Biomolecular Sciences, Universiti Putra Malaysia, UPM Serdang, Selangor 43400, Malaysia

**Keywords:** *Achillea biebersteinii*, apoptosis, cancer, green biosynthesis, nano-silver

## Abstract

Silver nanoparticles (Ag-NPs), the most popular nanoparticles, possess unique properties. *Achillea biebersteinii* is a plant of the *Asteraceae* family rich in active antitumor components. The aim of this research was the characterization and investigation of the cytotoxic properties of Ag-NPs synthesized using *A. biebersteinii* flower extract, on a human breast cancer cell line. The Ag-NPs were synthesized after approximately 180 min of reaction at 40 °C, then they were characterized by UV-visible spectroscopy, Fourier transform infrared spectroscopy (FTIR), transmission electron microscopy (TEM) and dynamic light scattering (DLS). The anti-apoptosis effect of Ag-NPs on the MCF-7 cell line was investigated by MTT assay, DAPI and acridine orange staining and caspase activity. The transcriptional expression of bax, bcl-2, caspase-3, -8 and -9 were also evaluated by RT-PCR. The TEM images revealed that the Ag-NPs morphology had a different shape. The DLS indicated that the average hydrodynamic diameter of the biosynthesized Ag-NPs was around 12 nm. By UV-visible spectroscopy the strongest absorbance peak was observed at 460 nm. The FTIR results also showed interaction between the plant extract and Ag-NPs due to the similarity in the peak patterns. The EDS results showed that Ag-NPs display an absorption peak at 3 keV, indicating the presence of the element silver. The Ag-NPs caused a dose-dependent decrease in cell viability, fragmentation in nucleic acid, inhibited the proliferation and induction of apoptosis on MCF-7 by suppressing specific cell cycle genes, and simulation programmed cell dead genes. Further investigation is required to establish the potential of this novel and promising approach in cancer therapy.

## 1. Introduction

*Achillea biebersteinii*, the herb known as yarrow, is a member of the Asteraceae family and has been used in traditional medicine for hundreds of years in many countries [1]. Yarrow has been traditionally used to treat diseases such as inflammatory and spasmodic gastrointestinal disorders, hepatobiliary complaints and overactive cardiovascular and respiratory ailments [2]. Furthermore, this medicinal plant is used as an appetite-enhancing agent because of its bitter taste, and has also been used in wound healing [3]. The aerial parts of *A. biebersteinii*, especially its flowers, are generally applied as aqueous or alcoholic extracts. Different researchers have reported the antioxidant, antimicrobial, antitumor and antifungal activity of *A. biebersteinii* extracts [4]. In recent years, many studies are being conducted on noble metallic nanoparticles, in particular Ag, Pt, Au and Pd. Nanomaterials can be synthesized by different methods, including chemical methods, physical methods and biological ones. Synthetic methods involving various chemicals may lead to the presence of toxic chemical species adsorbed on the NP surface, that may have adverse effects in biological applications [5].

The use of environmentally benign materials like plant leaf extracts, bacteria and fungi for the synthesis of nanoparticles, such as gold and Ag-NPs, has received increasing attention in recent years [6,7]. The green synthesis of nanoparticles offers numerous benefits of eco-friendliness, cost effective and compatibility for pharmaceutical and biomedical applications as they do not use toxic chemicals in the synthesis protocols [8]. Ag-NPs are attractive in various biological and pharmaceutical fields, such as ultrasensitive detection and imaging methods for bio-reorganization events, because Ag-NPs have unique optical properties (*i.e.*, surface plasma resonance absorption and resonance light scattering) and great biocompatibility. The use of Ag-NPs is a promising method for the treatment of a wide variety of diseases, including cancers. Hence, Ag-NPs have emerged with diverse medical applications including silver based dressings and silver coated medicinal devices, such as nano-gels and nano-lotions [9]. The green synthesis of Ag-NPs is important apropos of nanotechnology-based eco-friendly products [10]. The aim of this study was to investigate the anti-apoptosis properties on the MCF-7 (human breast cancer) cell line of Ag-NPs synthesized using *Achillea biebersteinii* flower extract.

## 2. Results and Discussion

### 2.1. Synthesis of Ag-Nanoparticles and Characterization

**Figure 1:**
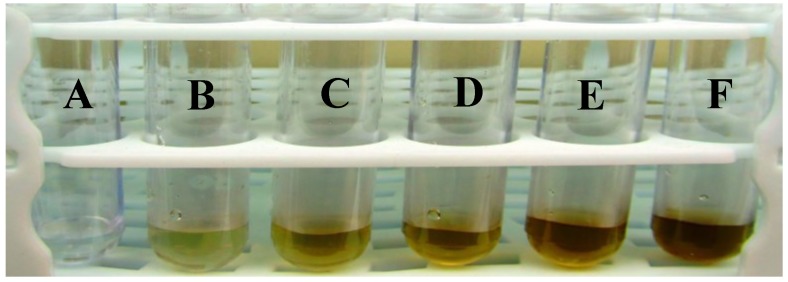
Visual appearance of vials containing A: Ag^+^ 5 mM; B: Flower extract *A. biebersteinii*; C: Ag^+^/A after 60 min; D: Ag^+^/A after 90 min; E: Ag^+^/A after 120 min; F: Ag^+^/A after 180 min.

**Figure 2:**
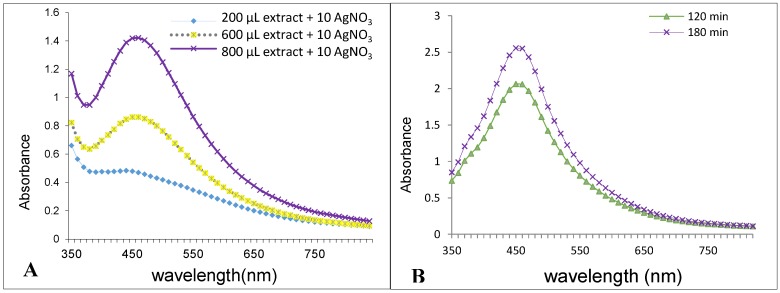
UV-visible spectra of the Ag-NPs synthesized with different volumes of plant extract at 180 min (**A**) and UV-visible spectra of the green synthesized Ag-NPs at different times (**B**).

It is well known that Ag nanoparticles exhibit a brown color in water; this is due to the excitation of surface plasmon resonance in the Ag-NPs. In the current study, the color change from colorless to brown of a mixture containing only AgNO_3_ solution and *A. biebersteinii* extract occurred within 180 min at 40 °C. The highest color intensity was observed in a solution containing 10 mL of silver nitrate (5 mM) and 0.8 mL of plant extracts (10:0.8 *v*/*v*) (Figure 1). UV-Vis spectroscopy is commonly used to examine the size and shape of nanoparticles in aqueous suspensions [11]. It is also well known that solutions containing Ag-NPs produce a characteristic absorption peak in the range of 420 to 480 nm [12]. Therefore, UV-Vis spectra of Ag-NPs were recorded over the wavelength range from 350 to 800 nm. The sharpest absorption peak for Ag-NPs was observed in 460 nm, after 180 min with ratio of 0.8 mL of *A. biebersteinii* extract and 10 mL AgNO_3_ (Figure 2). 

It is observed that the intensity of SPR bands increased as the reaction time progressed from 0–180 min. The synthesized Ag-NPs from flowers of *A. biebersteinii* extracts were observed to be stable in the solution as deposits were not formed after 2 weeks (Figure 2A,B).

**Figure 3:**
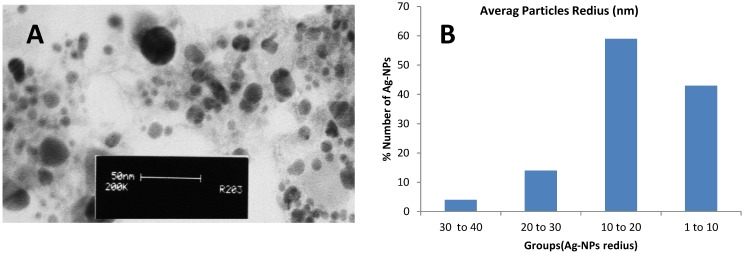
Transmission electron microscopy image of the green synthesized silver nanoparticles (**A**); Particle size distribution histogram of Ag-NPs (**B**).

TEM is a powerful method to determine the size of nanoparticles [13]. Therefore, the morphology and size of green synthesized Ag-NPs were studied using TEM. Figure 3A provides an overview of the range of sizes and distribution of the Ag-NPs, showing spherical and pentagonal shapes and according to the results from Ag-NP diameter measurements using image J software, a size range of 10 to 40 nm. Figure 3B shows the particle size distribution histogram of the Ag-NPs determined from the TEM image.

**Figure 4:**
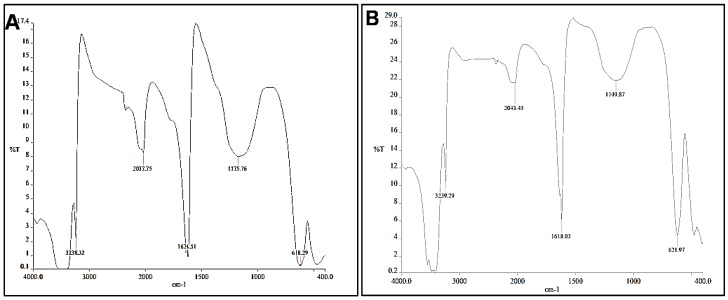
FTIR spectra of bio-synthesized Ag-NPs (**A**); *A.biebersteinii* extract (**B**).

The FT-IR spectra recorded from the *A. biebersteinii* flower extract and also the synthesized Ag-NPs are shown in Figure 4. Before recording the FT-IR, the Ag-NPs were centrifuged repeatedly to ensure any free plant extract material was removed from solution. As can be seen both spectra are similar. The wide band in the range of 3000–3400 cm^−1^ arises from the -NH_2_ and -OH groups in protein molecules [14], so it can be conformed that proteins are present in the *A. biebersteinii* flower extract that acted as a reducing agent. Moreover the carbonyl group of amino acid residues in proteins has a strong binding ability to metal nanoparticles and formed a coating layer on the surface of the Ag-NPs, which prevents Ag-NPs agglomeration in the aqueous medium. The FT-IR spectra also revealed the presence of C-O and O-H (phenolic compound) functional groups on the surface of Ag-NPs which also can be effective in Ag ion reduction [14].

Many species of *Achillea* contain more than 100 components, such as flavonoids and other phenolic compounds [15], and previous reports have shown that phenolic compounds can reduce Ag ion to silver nanoparticles [16]. Therefore, phenolic compounds present in plant extracts can be effective for the reduction of Ag ions and the production of Ag-NPs. Moreover, it was reported that proteins also can lead to the reduction of metal ions, as they bind to the nanoparticles either through free amine groups or cysteine residues in the proteins and act as both reducer and covering agent [11].

**Figure 5:**
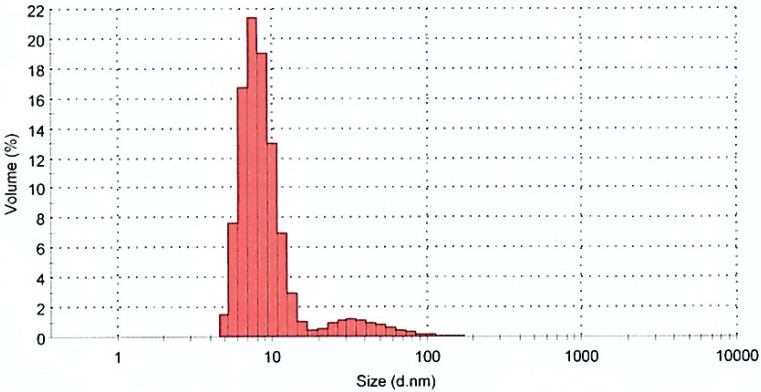
Particle size distribution of biosynthesized Ag-NPs.

The application of the dynamic light scattering (DLS) method for determining the exact size distribution of colloidal metallic nanoparticles has been discussed in earlier research [17]. However, in our study, as can be seen in Figure 5, the DLS results showed that the distribution range of particles was from approximately 8 nm to 100 nm. Thus the average size of the Ag-NPs was found to be 12 nm. According to Figure 3, it is confirmed that the sample contains various sizes of nanoparticles, and the average size agrees with the result obtained from the TEM analysis (13.64 nm). The difference in reported Ag-NPs size is due to different measurement techniques, also TEM image represent a small portion of the sample but DLS provides a manifestation the entire sample size. However in any case, the numbers obtained from both techniques are very similar and close.

### 2.2. Cell Cytotoxicity

**Figure 6:**
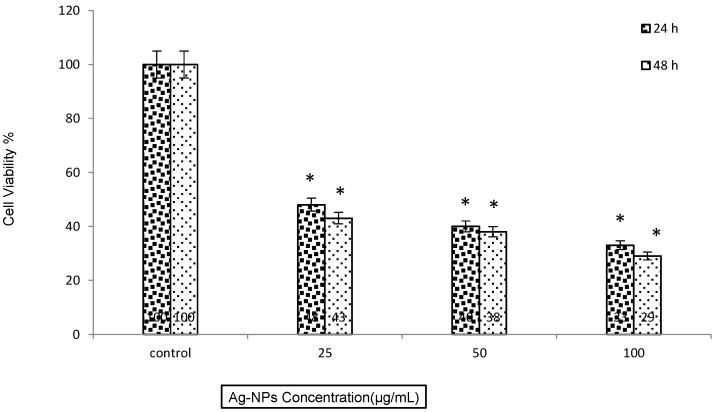
MTT assay results from treatment MCF-7 cell with various Ag-NPs concentration in 24 h and 48 h, Mean ± SD, * *p* ˂ 0.05.

The toxicity potential of the synthesized Ag-NPs on human breast cancer cells has been examined using the MCF-7 cell line (Figure 6). The cells were exposed to various concentrations of Ag-NPs for 24 h and 48 h then the toxicity effects of Ag-NPs assessed using the MTT assay. The MTT results showed that the Ag-NPs decreased cell viability dose and time dependently. The inhibitory concentration (IC_50_ value) was 20 µg/mL after 24 h of cell treatment. It is similar to IC_50_ that reporting with other researchers [8,11,18].

### 2.3. Morphological Change Observations Using Acridine Orange (AO) and DAPI Staining and Bright Field Microscopy

**Figure 7:**
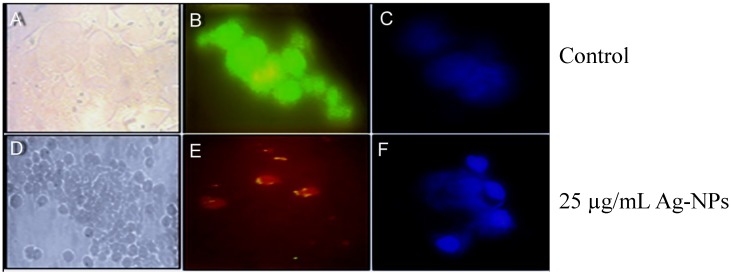
Comparing morphological change assess in MCF7 cells that treatment with Ag-NPs and Control (**A**) light microscopy image of control; (**B**) AO staining in control group; (**C**) DAPI staining of control group; (**D**) Ag-NPs caused significant cell death; (**E**) staining with AO, 25 µg/mL Ag-NPs caused 50% cell death; (**F**) DAPI staining shows fragmentation of nucleic acid after treatment (24 h).

**Figure 8:**
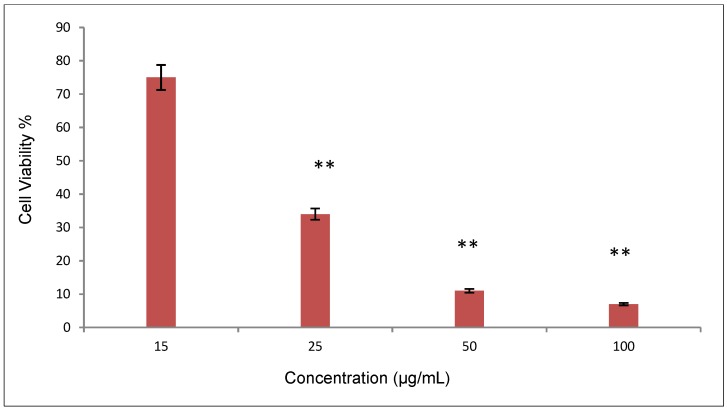
Percentage of live and dead cells calculated from AO/EB staining, ** *p* < 0.001.

Some fluorescent stains including Hoechst, DAPI, ethidium bromide (EB), and AO label DNA and allow easy visualization of the nucleus in cells. To observe any nuclear morphological changes induced by Ag-NPs, DAPI staining was used. DAPI binds with the minor groove of double-stranded DNA, and in this state DAPI fluorescence increases approximately 20-fold. AO is a fluorescent dye and can stain both dead and alive cells, but EB can only stain dead cells that have lost membrane integrity. Live cells are exited uniformly green. Apoptotic cells are also permeable to EB and stain orange, while necrotic cells stain orange-red. AO/EB also used for the detection of morphological changes in treated and untreated cells. For this purpose MCF-7 cells were treated with IC_50_ concentrations of Ag-NPs. When compared with the untreated cells, morphological changes were observed in the MCF-7 cells treated with Ag-NPs. The most recognizable morphological changes in the treated groups were cytoplasmic condensation, cell shrinkage and aggregation of nuclear chromatin into dense masses (Figure 7). DAPI also proved the Ag-NPs induced DNA fragmentation in cells treated with 25 µg/mL Ag-NPs. DNA breakdown is a hallmark of apoptosis. The nucleic acid morphological changes were examined by DAPI staining, which clearly showed nucleic acid fragmentation caused by the treatment with Ag nanoparticles, which confirmed the results of AO staining (Figure 7F). Staining cells with AO enables us to distinguish and quantitatively determine the percentage of dead, viable, apoptotic and necrotic cells after treatment with Ag nanoparticles. At 24 h, the percentage of apoptotic cells increased from 0% in the control culture to 50% in cells treated with 25 µg/mL Ag nanoparticles and the proportion of the late apoptotic (yellow)/necrotic (red) cells increased significantly from 1% to 45% while the number of live ones (green) decreased dramatically (Figure 8).

Several pathological syndromes, such as liver failure, stroke and heart attack, are associated with the abrupt death of tissue or organs as a result of apoptotic deregulation. In addition, the survival of abnormal cells, due to aberrant apoptosis, may lead to tumorigenesis [19,20]. Apoptosis is commonly altered in cancerous cells, and those cells have the ability to evade the apoptotic cascade. The Ag-NPs synthesized using *Achillea biebersteinii* extract significantly increased the programmed cell death in the treated MCF-7 cells. Ag-NPs inhibited the proliferation of MCF-7 cells dose and time dependently, which demonstrated that the Ag-NPs prepared using *Achillea biebersteinii* have great promise as an anti-tumor agent. The findings of this study indicated the potential for *Achillea biebersteinii* in drug development against cancer.

### 2.4. Caspase Activity

**Figure 9:**
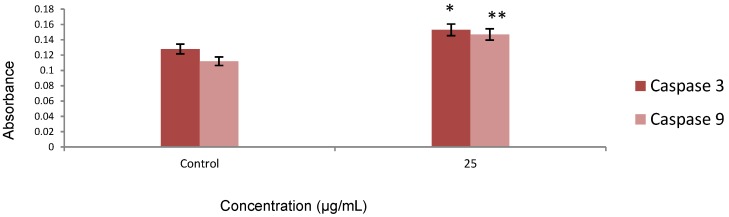
The activity of caspase-3 and caspase-9 was increased after 24 h treatment, which indicates significant apoptosis * *p* < 0.05, ** *p* < 0.001, Data are presented as mean ± SD.

The apoptotic effect of the synthesized Ag-NPs was also examined by determining caspase-3 and caspase-9 relative to the varying concentration of protein content for cells treated with the IC_50_ of Ag-NPs for 24 h. Our results showed that caspase activity increased in treated cells, as shown in Figure 9. Caspase-3 and caspase-9 are members of the cysteine protease family, which have been identified as major regulators of programed cell death. These enzymes are involved not only in the initiation but also in the execution phase of apoptosis by cleaving more than 400 substrates. This cleavage mediates the majority of the typical biochemical and morphological changes in apoptotic cells, such as cell shrinkage, chromatin condensation, and DNA fragmentation. Pathways to caspase-3 activation have been identified, which are either dependent on or independent of the release of the mitochondrial cytochrome c and caspase-9 function. Caspase-3 appears to amplify the caspase-9 initiation signals into full-fledged commitment to disassembly. Caspase-9 activates caspase-3 by proteolytic cleavage and caspase-3 then cleaves vital cellular proteins or other caspase [21]. Gurunathan *et al.* evaluated the potential toxicity of biologically synthesized Ag-NPs in MDA-MB-231 human breast cancer cells. They indicated an increased level of caspase-3 activation in the treated cells. This result coincides with our finding [22]. We tested the effect of Ag-NPs on the cascade of caspases that are crucial initiators or effectors in the cell death pathways. The enzymatic activity of caspase-8 was unchanged after 24 h of incubation at all different concentrations (data not shown). A significant activation of caspase-3 and -9 occurred at 25 µg/mL Ag-NPs after 24 h of incubation.

### 2.5. Gene Expression by RT-PCR

**Figure 10:**
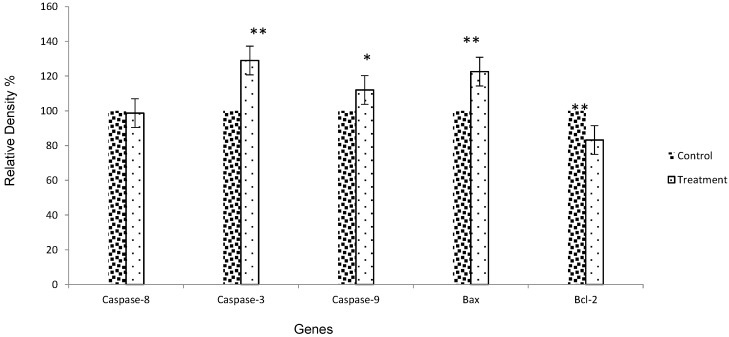
Diagram of apoptosis gene expression, * *p* < 0.05, ** *p* < 0.001.

Apoptosis or programmed cell death is a gene-regulated phenomenon, which is important in both physiological and pathological conditions. The important regulatory mechanisms of apoptosis include death receptors, activation of caspases, mitochondrial responses and Bax, and the regulation of Bcl-2 gene expression [23]. The products of BCL-2 and BAX genes heterodimerize or homodimerize and the relative levels of the available dimerization partners shift the balance of cell fate in favor of either viability or cell death [24]. We demonstrated an inverse relationship between apoptosis and BCL-2 in the MCF-7 cell line after treatment. There was also an inverse relationship between the BCL-2 and BAX gene expression. In this study, we examined the inhibitory effects of Ag-NPs on the downregulation of Bcl-2 gene expression, which was remarkable, with 17.8% reduction (*p* < 0.001) (Figure 10). Bax gene expression was significantly increased after treatment with Ag-NPs (*p* < 0.001). As our results showed, the expression of Bax and Bcl-2 gene expression could be regulated differently by Ag-NPs, which suggests that a balance in the expression of these genes and their proteins might be involved in the control of the apoptosis process. The results of electrophoresis in agarose gel by looking at a single sharp band was detected, which did not reveal any significant reduction (*p* > 0.05) in the caspase-8 gene expression. This result indicated that caspase-8 was not activated in response to the Ag-NPs treatment, and that the extrinsic pathway was not involved. On the other hand, Ag-NPs induced caspase-3 activation and apoptosis, which may reveal a mitochondrial pathway. In our study, caspase-9 processing was coincident with the dramatically reduced activation of caspase-3 with *p* < 0.005 and *p* < 0.001, respectively, which indicated that the highest activity of caspases was implicated in the mitochondrial apoptosis pathway after Ag-NPs treatment. Data from the present research conformed with previous studies, which detected the role of apoptotic proteins, such as caspases and some apoptotic biomarker genes like Bax, Bcl-2, Apaf-1 and PARP [25,26].

## 3. Experimental Section

### 3.1. Ag-NP Biosynthesis and Characterization 

The whole flowers of *Achillea biebersteinii* were collected from Iran (Khorasan, Masshad, May 2013). The identity of the plant material (voucher specimen number 34516) was confirmed by a plant taxonomist from the Herbarium Division of the College of Ferdowsi University of Mashhad. The fresh flowers of *Achillea biebersteinii* were washed thoroughly three times with double distilled water, and air-dried in the shade at room temperature for two weeks, following which they were powdered in a mixer and used for extraction. They were chopped into small pieces and the resultant pieces (5 g) were soaked in deionized water (100 mL) and heated to boiling point for at least 10 min. Finally, the solution was filtered through Whatman filter paper (No. 1) and stored at 4 °C for further use. For the preparation of Ag-NPs, a solution of 10 mL of AgNO_3_ solution (5 mM) with 100 to 1000 µL of *A. biebersteinii* flowers extract was used, which was warmed to 40 °C until the color of the solution slowly turned from yellow to dark brown (the process carried out in 40 °C and pH = 7). For characteristization, the UV-visible absorption spectra of the prepared nanoparticles were measured using a UV-vis spectrophotometry system (Epoch, Biotek, Winooski, VT, USA) at different times and concentrations over a wavelength range of 350–800 nm. The morphology and size of the synthesized Ag-NPs were recorded using a field transmission scanning electron microscope (Hitachi, Tokyo, Japan). Fourier transform infrared (FT-IR) spectra (Perkin Elmer, Walthman, MA, USA) were used to identify the functional groups of the active components based on the peak value in the region of infrared radiation. The product was then centrifuged at 9000 rpm for 30 min and dried. The zeta-potential of Ag-NPs in water were evaluated by means of a zetasizer, using CAD, zeta compact, Les Essarts-le-Roi, France. The average size and stability of the nanoparticles were determined using DLS analysis. Finally, the synthesized Ag-NPs were dried, drop coated on to carbon film, and tested using EDS analysis (XL 30; Philips, Eindhoven, The Netherlands). 

### 3.2. Cell Culture and Apoptosis Assay

Human breast cancer cell lines (MCF-7) were cultured in RPMI 1640 medium supplemented with 10% fetal bovine serum, 100 units/mL penicillin, and 100 µg/mL streptomycin, incubated at 37 °C under a 5% CO_2_/95% air atmosphere.

#### 3.2.1. MTT Assay

MCF-7 cells (5000) were seeded onto a 96-well plate and incubated overnight in 95% humidity, 5% CO_2_ at 37 °C. After 24 h, to ensure cell adhesion and confluence in the wells, the medium was replaced with a fresh one containing Ag nanoparticles in different concentrations (1–100 µg/mL) for 24 h and 48 h. The effects of Ag-NPs on cell viability were estimated by a MTT assay using 3-(4,5-dimethylthiazol-2-yl)-2,5diphenyl tetrazolium bromide; 10 µL of MTT solution was added to each well and the plate was incubated for two hours in dark conditions, then 100 µL DMSO was added to solubilize the MTT. In the last part, the absorbance of each well was measured at 570 nm with a microplate spectrophotometer. Concentrations of Ag-NPs showing 50% reductions in cell viability (*i.e.*, IC_50_ values) were calculated, and the percent mitochondrial activities of treated cells against untreated cells (as control group) were determined (Figure 7).

#### 3.2.2. DAPI (4,6-Diamidino-2-phenylindole dihydrochloride) Staining

The treated MCF-7 cells were fixed with 4% paraformaldehyde (20 min at 4 °C and then 5 min at RT), which were previously washed with PBS. Then, the cells were washed with Triton X100 0.4%, 20 min in RT and washed with cold PBS twice. Finally, the cells were dyed with DAPI. For nuclear staining DAPI (1 µg/mL) was added for 60 s in the dark and washed. The stained images were captured by means of a fluorescent microscope with appropriate filter (Figure 7).

#### 3.2.3. Acridine Orange (AO) and Ethidium Bromide (EB) Staining

A dye mixture with an equal ratio of acridine orange (AO) and ethidium bromide (EB) was prepared and mixed with cell suspension and put on a clean microscopic cover slip. After incubation for approximately 2–3 min, the cells were visualized under a fluorescence microscope at 40× magnification via a special filter at 510–590 nm. The percentage of apoptotic cells was calculated using the following formula [12]:

(1) $$\text{\% of apoptotic cells}=\frac{\text{Total number of apoptotic cells}}{\text{Total number of normal and apoptotic cell}}\times \;100$$

#### 3.2.4. Caspase-3 and Caspase-9 Assay

The activity of caspase-3 and caspase-9 was measured using the Abcam Kit colorimetric protease assay following the protocol provided by the manufacturer. Briefly, the cells were treated with Ag-NPs for 24 h. Cell pellets containing 1–5 × 10^6^ cells were suspended in 50 µL of chilled Cell Lysis Buffer and the cells were incubated on ice for 10 min and centrifuged for 1 min using a micro-centrifuge (10,000× *g*). Then, the protein concentration was examined using the Biuret method: dilute 50–200 µg protein with 50 µL Cell Lysis Buffer, 2× Reaction Buffer, DTT to each well and 4 mM DEVD-p-NA substrate. Finally, the samples were read at 400 or 405 nm in a micro-titer plate reader (Epoch, Biotek, Winooski, VT, USA). The fold-increase in caspase-3 activity could be determined by comparing these results with the level of the un-induced control.

#### 3.2.5. RT-PCR

The changes in the expression of bax, bcl-2, caspase-3, -8 and -9 genes were analyzed using RT-PCR. Briefly, the total RNA of the treated MCF-7 cells was isolated by the High Pure RNA Isolation kit according to the manufacturer’s protocol (Roche, Mannheim, Germany), then the cDNA was synthesized using the Fermentas Kit, incubated at 65 °C for 5 min, followed by the addition of the RT premix. The temperature of the synthesis was according to the protocol: incubation at 65 °C for 5 min, followed by the addition of components: 5× Reaction Buffer, RiboLuck RNase Inhibitor, dNTP Mix and Revert Aid M-MuLV RT. Then, incubation at 42 °C for 60 min and 70 °C for 5 min. Finally, 2 µL of the cDNA produced was added to 10 µL Taq Premix and the appropriate forward and reverse primers. Ultimately, RT-PCR was performed with 1 cycle at 94 °C/5 min, 30 cycles at 94 °C/30 s for denaturation, 56 °C/30 s for annealing, 68 °C/45 s for extension and 1 cycle 5 min at 72 °C according to the Pars-Tous manufacturer’s protocol. The primers used are shown in Table 1.

**Table 1:** Sequence of primer.

Gene	Forward Primer	Reverse Primers
**Beta Actin**	5′ CCC GCC GCC AGC TCA CCA TGG 3′	5′ AAG GTC TCA AAC ATG ATC TGG GTC 3′
**Bax**	5′ TTTGCTTCAGGGTTTCATCCA 3′	5′ CTCCATGTTACTGTCCAGTTCGT 3′
**Bcl-2**	5′ CATGTGTGTGGAGAGCGTCAAC 3′	5′ CAGATAGGCACCCAGGGTGAT 3′
**Caspase-3**	5′ TATGGTTTTGTGATGTTTGTCC 3ꞌ	5′ TAGATCCAGGGGCATTGTAG 3′
**Caspase-8**	5′ CTACCAACTCATGGACCACAG 3′	5′ GTGACTGGATGTACCAGGTTC 3′
**Caspase-9**	5′ TACAGCTGTTCAGACTCTAGTA 3′	5′ AAATATGTCCTGGGGTAT 3′

The PCR products were observed by electrophoresis in a 2% agarose gel and read using a UV-detector (Uvitec, Cambridge, UK). Finally, the percentage of relative density for each gene was measured using Image J software.

#### 3.2.6. Statistical Analysis

Statistical evaluation of the data was performed using one-way analysis of variance (ANOVA), Tukey test used for multiple comparisons as a post-test with the help of SPSS software. The results are shown as mean ± SD and *p* < 0.05 accepted as the minimum level of significance.

## 4. Conclusions

Previous findings confirm the cytotoxic properties of Ag-NPs, and suggest that they may be a cost-effective alternative measure in the field of cancer therapeutics [25]. In the present study Ag-NPs were synthesized using *Achillea biebersteinii* flower extract both as reducing agent and capping agent. This design has several advantages which provide for the combined use of two anti-cancer agents (*Achillea biebersteinii* flower extract and Ag-NPs) at low cost and in a short time. In summary, our data indicated that the biosynthesized Ag-NPs induced apoptosis on MCF-7 cell line, which was confirmed using AO/Et and DAPI staining. Caspase-3 and -9 activities showed that the cell death involved a caspase dependant intracellular pathway. The Ag-NPs downregulated the anti-apoptotic genes of the bcl-2 family and unregulated the pro-apoptotic members, such as bax. Biosynthesized Ag-NPs from *Achillea biebersteinii* flower extract could be considered a potential chemotherapeutic agent in the treatment of breast cancer.
